# Supplementary material for: Parsimonious neural networks learn interpretable physical laws
Source: Sci Rep. 2021 Jun 17;11:12761. doi: 10.1038/s41598-021-92278-w (PMC8211802; doi:10.1038/s41598-021-92278-w)
Supplement: Supplementary file 1 — Supplementary Files. [file 41598_2021_92278_MOESM1_ESM.docx]

[Supplementary Material]

Parsimonious neural networks learn interpretable physical laws

Saaketh Desai and Alejandro Strachan

School of Materials Engineering and Birck Nanotechnology Center

Purdue University, West Lafayette, Indiana, 47907 USA

## S1. Description of training data to discover integration schemes

The data used to train the feed forward neural networks (FFNNs) and the parsimonious neural networks (PNNs) is a set of trajectories generated using the LAMMPS software package [1], a popular choice to perform molecular dynamics simulations. We generate trajectories of a two-particle system where interactions between the atoms are described by a Lennard-Jones potential with parameters $\epsilon$ = 0.5 eV and $\sigma$ = 2.5 Å. The system consists of one particle at the origin and the other particle fixed at x = 1.12$\sigma$ (the y and z coordinates are fixed to be 0). The simulation cell is arbitrarily chosen to be cubic with a side length of 80$\sigma$ to avoid interactions between periodic images of particles.

Four different trajectories were generated under the microcanonical (NVE) ensemble, with different initial velocities for the atom at origin, corresponding to total energies of -0.999$\epsilon$, -0.895$\epsilon$, -0.797$\epsilon$ and -0.733$\epsilon$, using a timestep of 0.011962$\sqrt{\frac{\epsilon}{m\sigma^{2}}}$. The equations of motion are integrated using the velocity Verlet algorithm and each trajectory contains 100,000 snapshots of atomic coordinates, velocities and forces at each step, for a total of 400,000 snapshots. We consider this highly accurate trajectory to be our baseline, from which we sample frames at different frequencies to obtain trajectories at different timesteps. So, to sample a trajectory with timestep 0.11962$\sqrt{\frac{\epsilon}{m\sigma^{2}}}$ , we sample every 10^th^ snapshot from each trajectory. This amounts to 40,000 data points consisting of input and output arrays, with the input array consisting of position, velocity and force at time $t$, while the output array consisted of position, velocity and force at time $t+\Delta t$ (i.e., one step later). The data is split into training and validation sets in an 80:20 ratio, with an additional test set of 10,000 points was generated independently at an energy of -0.884$\epsilon$. For the damped dynamics cases, a frictional force proportional to negative the velocity is added, with frictional coefficient γ = 0.004 eVps/Å^2^. Note that the force sub-net used in the PNN is not updated with the frictional force, meaning that the only way for the PNN to learn about the damping is through the training data trajectories.

While all quantities are mentioned in Lennard-Jones units, in practice we use a different unit system, with positions in pm, time in fs and energy in eV. This ensures that the inputs to the network are of the same scale, where the standard practice of normalizing the data could lead to a loss of interpretability in a general case. The training data is available on nanoHUB [2].

## S2. Standard feed forward neural network

As a baseline, we train a standard feed forward neural network (FFNN) and evaluate its performance as an integrator. This network consists of three hidden layers consisting of 20, 100 and 20 neurons respectively, with the rectified linear (‘*relu’)* activations applied to the hidden layers and linear activations applied to the output layer. We use mean squared error (MSE) as the loss function and the Adam optimizer [3] with a learning rate of 10^-3^. The network is trained using the Keras package [4] and the training data and models are available on nanoHUB [2]. We find that the FFNN is inadequate as a surrogate integration scheme, with significant energy drifts and a lack of time reversibility, see Figure S1. The root mean squared errors (RMSE) on the position and velocity, across the training, validation and testing sets are (1.48x10^-5^, 9.73x10^-5^), (1.54x10^-5^, 9.98x10^-5^) and (1.59x10^-5^, 9.94x10^-5^) respectively. These errors serve as a baseline for any subsequent networks, with any successful integration scheme necessarily having errors lower than the feed forward NN.


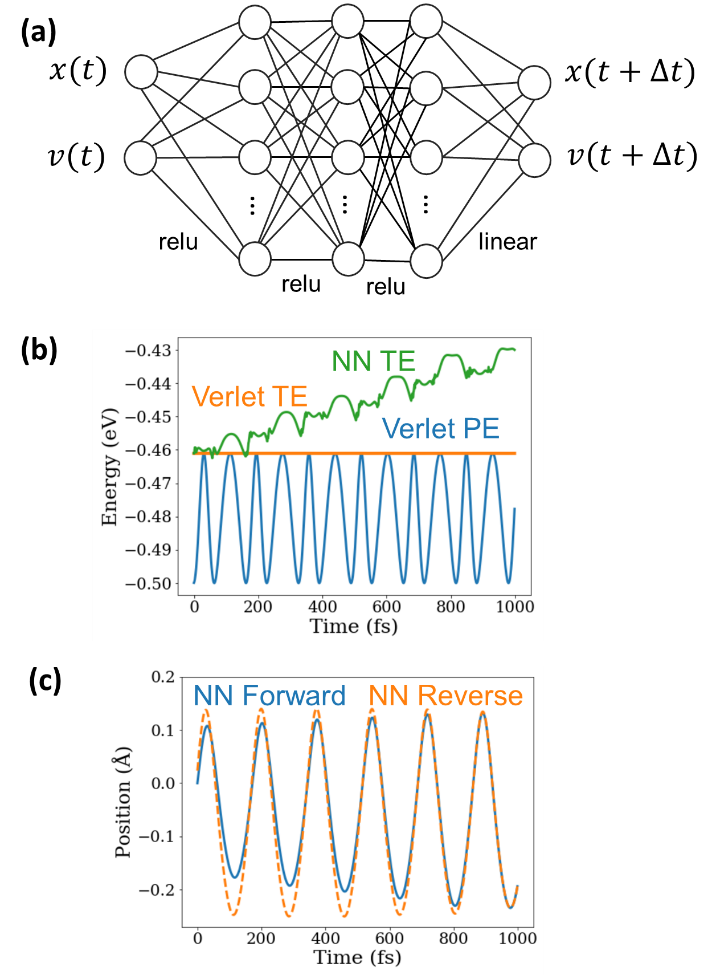


*Figure S1: (a) Standard feed forward neural network, attempting to predict positions and velocities one step ahead (b) Energy drift for the feed forward NN compared to the Verlet algorithm (c) Forward and reverse trajectories generated by the feed forward NN, showing the lack of reversibility*

## S3. Feed forward neural network with a force sub-net

Having established that a standard feed forward neural network performs poorly as integrator (see Figure S1), we seek to establish whether a standard feed forward neural network with a force sub-net built in can perform well as an integrator. The force sub-net is a two-layer network (with 10 neurons in each layer) that attempts to predict the force given a position as input, as shown in Figure 1 of the manuscript. The hidden layers of this network have ‘tanh’ activations and the network is trained using the same protocol as the FFNN. We thus build a network as shown in Figure S2(a) and train the network, again using the same protocols used for the FFNN. We find that the network still performs poorly as an integrator with RMSEs on the train, validation and test sets for the position and velocity of (1.60x10^-5^, 3.84x10^-4^), (1.76x10^-5^, 4.95x10^-4^) and (1.56x10^-5^, 4.38x10^-4^). Figure S2(b) and S2(c) document the poor energy conservation and reversibility.


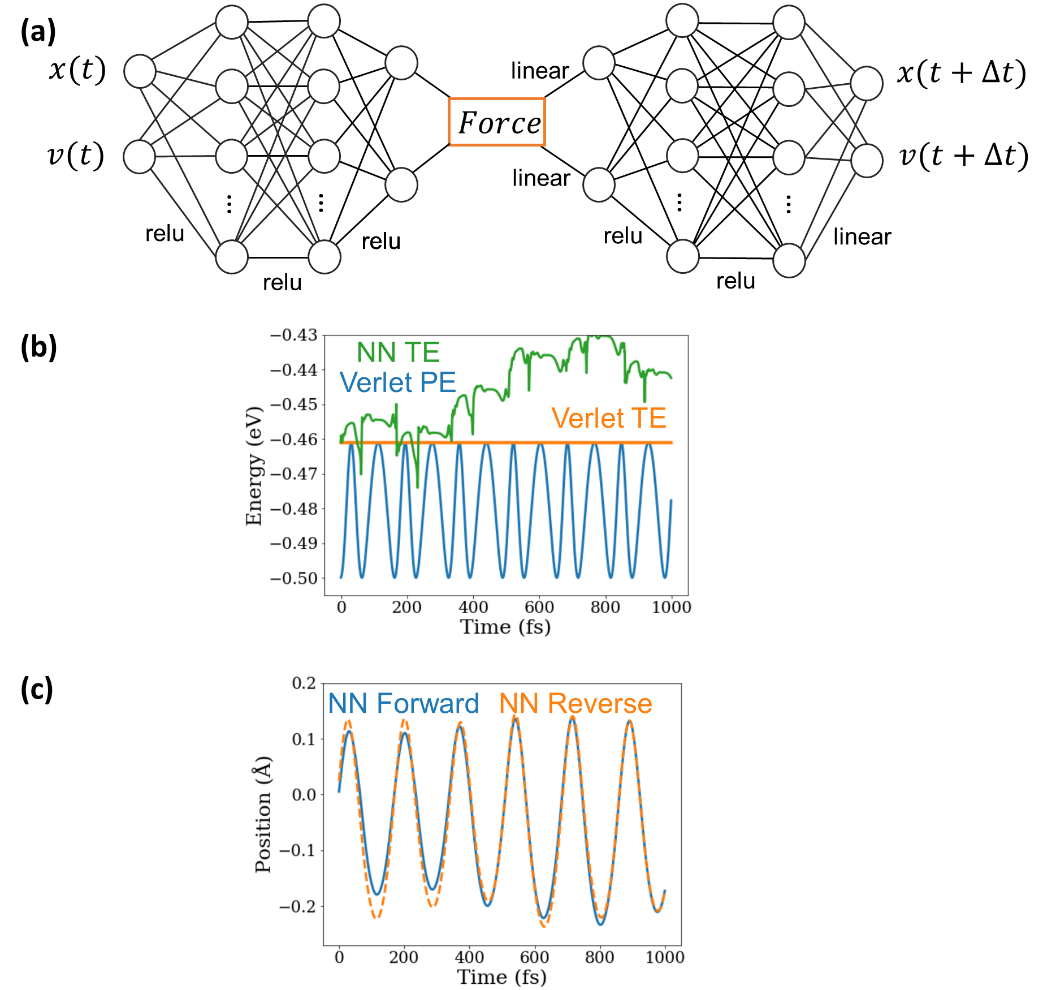


*Figure S2: (a) Feed forward neural network with a fixed force sub-net built in (b) Energy conservation (TE: total energy) and (c) reversibility are poor even for this network*

## S4. Detailed description of the genetic algorithm objective function

The genetic algorithm is designed to find the most parsimonious description of our data, and thus favors: i) linear activation functions over non-linear ones and ii) fixed weights with simple values (0, ½, 1, 2, $\frac{\Delta t}{2}$, $\Delta t$, and 2$\Delta t$, with $\Delta t$ the timestep) over optimizable weights. We define a fitness value for each individual network that is trained by the genetic algorithm as follows:

$$F=f_{1}\left( E_{test} \right)+\Sigma_{i=1}^{6}w_{i}^{2}+\Sigma_{j=1}^{20} f_{2}\left( w_{j} \right)$$

Where $-F$ is the fitness ascribed to each individual (lower F is fitter), $E_{test}$ is the mean squared error on the test set, $w_{i}$ is the score associated with the *i^th^* activation function and $w_{j}$ is the score associated with the *j^th^* weight.

This objective function consists of three terms, one corresponding to the ability of the network to describe the test dataset, defined as follows:

$$f_{1}\left( E_{test} \right)=10{log}_{10} \left( E_{test} \right)$$

where the logarithmic function converts the wide range of errors possible into values comparable to the other terms of the objective function. The second term reflects the tendency of the genetic algorithm to favor linear activations over non-linear activations. The ‘linear’, ‘relu’, ‘tanh’ and ‘elu’ activation functions are assigned scores of 0, 1, 2 and 3 respectively, the second term thus favoring linear activations over non-linear ones. The third term favors fixed, simple weights over arbitrary values generated by having a trainable weight. A fixed weight value of 0 is assigned a score of 0, while other fixed weight values (½, 1, 2, $\frac{\Delta t}{2}$, $\Delta t$, and 2$\Delta t$, with $\Delta t$ the timestep) are assigned the score 1, and a trainable weight is assigned a score of 2, and each weight contributes to the objective function as follows:

$$f_{2}\left( w_{j} \right)=\left\{ \begin{aligned} 0,w_{j}=0 \\ 1,w_{j}=½, 1, 2, \frac{\Delta t}{2}, \Delta t, 2\Delta t \\ 2, w_{j}=trainable \end{aligned} \right.$$

## S5. Results from the genetic algorithm optimization

Given the fitness function in section S4, we can encode our network with 6 activations and 20 weights as an individual of length 26. The first 6 genes of this individual can take values from 0 to 3, and the next 20 genes can take values from 0 to 7. We start with populations of 200 and 500 individuals, evolving them for 50 generations each to discover PNNs. We use a two-point crossover with a crossover probability of 0.5, and a custom mutation operation, where the first 6 genes are randomly mutated between 0 and 3 while the next 20 genes are randomly mutated between 0 and 7, with a mutation probability of 0.3. Individuals are selected for crossover using a tournament selection scheme with a tournament size of 10. For each individual, the fitness described above is evaluated, training the network using the functional API in the Keras package [4] if the network has trainable weights. We use the simple evolutionary algorithm described in Chapter 7 of [5], as implemented in the DEAP package [6]. For each population size, we perform five independent runs (using different seeds to initialize the initial population) to characterize the probability of successfully discovering an accurate integration scheme. The evolution of the fitness for the best individual in each generation is shown in Figure S3. The complete code for the genetic algorithm optimization can be found in a Jupyter notebook on nanoHUB [2].


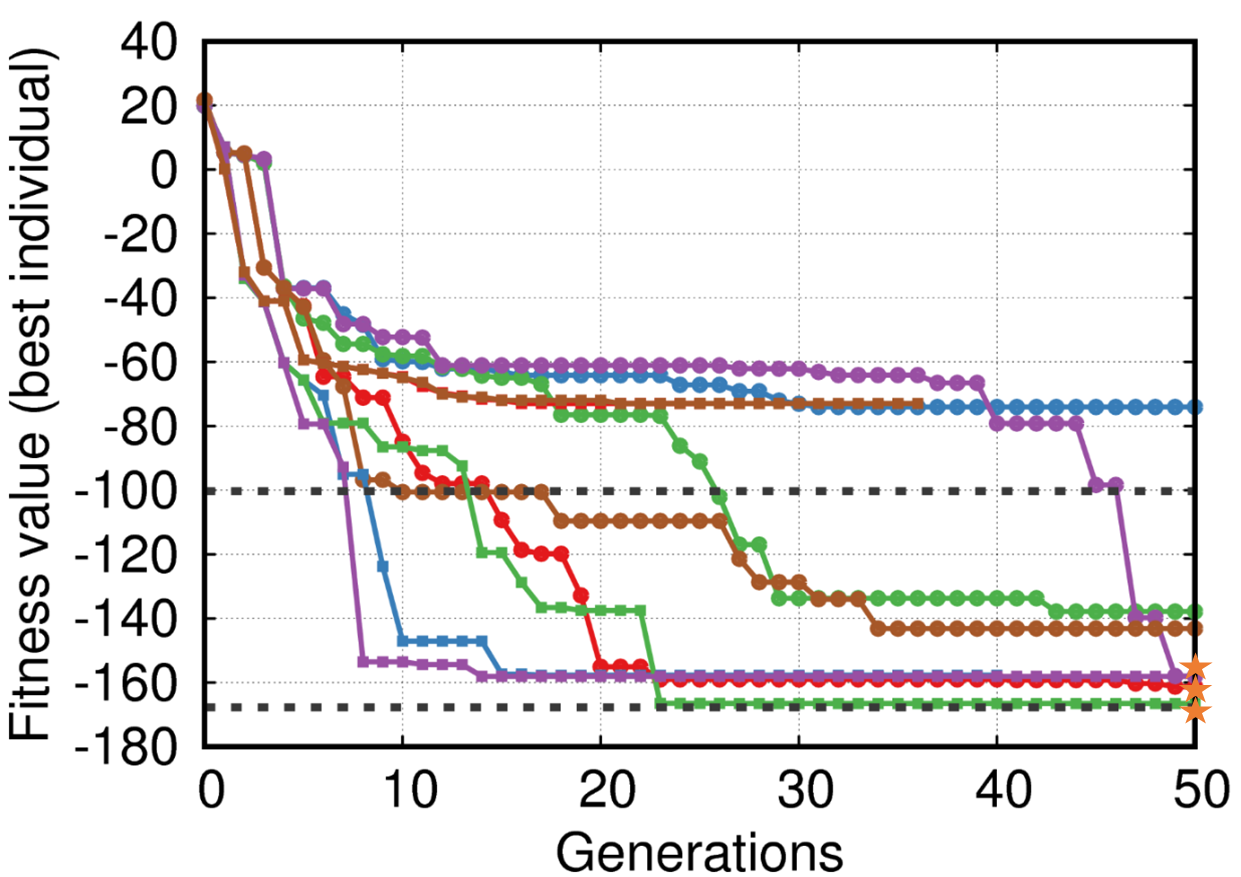


*Figure S3: Evolution of the fitness of the best individual in each generation. The colors represent different runs, while the black dashed lines represent the fitness value for the position Verlet algorithm and the Euler algorithm. Runs with a population size of 500 are marked with squares and runs with a population size of 200 are marked with circles. The orange stars represent the top 3 PNNs.*

## S6. Interpreting the Parsimonious Neural Network (PNN) Models

We describe here the interpretations of top 3 PNN models found. This interpretation is achievable due to the parsimonious nature of our networks, favoring linear activations over non-linear activations and integer or simple fractions for weights over arbitrary weights generated by training the network.

The best network discovered (PNN Model 1) can be shown as follows:


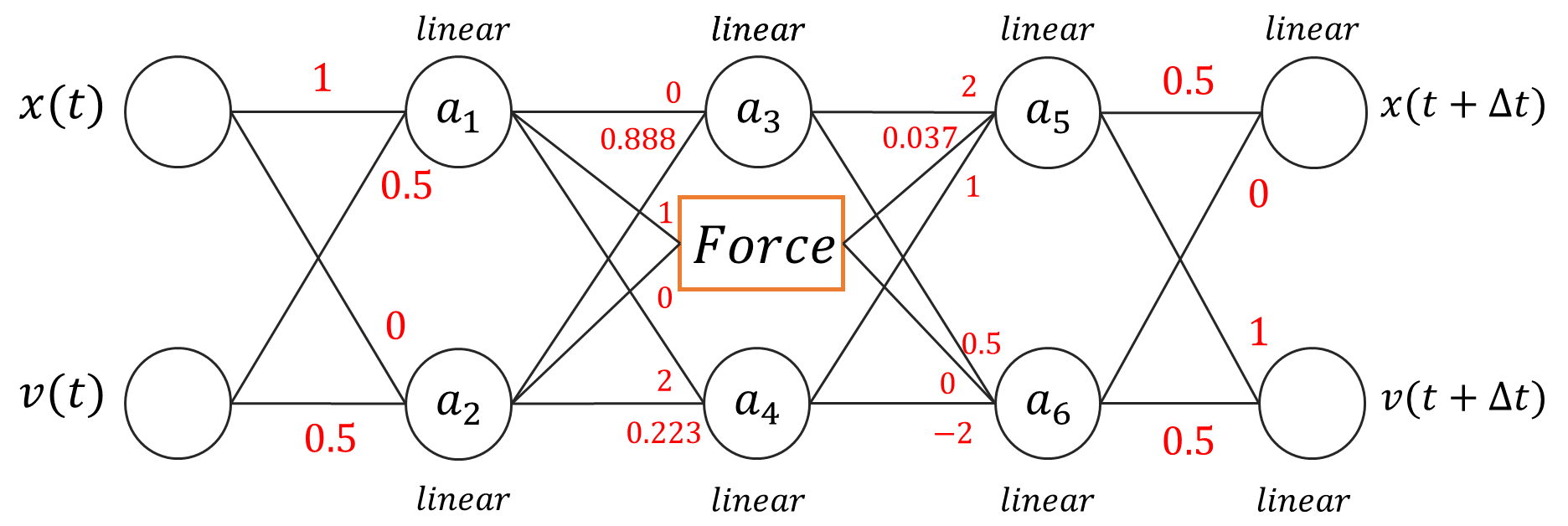


*Figure S4: Activation functions and weights for PNN Model 1*

The RMSE values on taking one step for the position and velocity are (1.88x10^-7^, 7.05x10^-7^), (1.89x10^-7^, 7.12x10^-7^), and (1.50x10^-7^, 4.73x10^-7^) respectively for the training, validation and testing set.

This network can be interpreted as:

| $a_{1}=x(t)+\frac{1}{2}v(t)$ | $a_{2}=\frac{1}{2}v(t)$ |
| --- | --- |
| $a_{3}=0.888a_{2}$ | $a_{4}=2a_{1}+0.223a_{2}$ |
| $a_{5}=2a_{3}+0.037f\left( a_{1} \right)+a_{4}$ | $a_{6}=\frac{1}{2}a_{3}-2a_{4}$ |
| $x(t+\Delta t)={\frac{1}{2}a}_{5}$ | $v(t+\Delta t)=a_{5}+{\frac{1}{2}a}_{6}$ |

We also see that the PNN models attempt to correct for the errors in the force sub-net, which could be the reason for the PNN models to learn the Verlet integration scheme with marginally different coefficients. Figure S5 shows the energy conservation for the Verlet integration scheme (using the force sub-net) and PNN 1. The Verlet network consists of weights corresponding to the position Verlet integration scheme, and we find that the network predicts a total energy slightly different from the true total energy, the discrepancy originating from an imperfect force sub-net. PNN 1 attempts to correct this discrepancy, resulting in better predictions of the total energy.

*
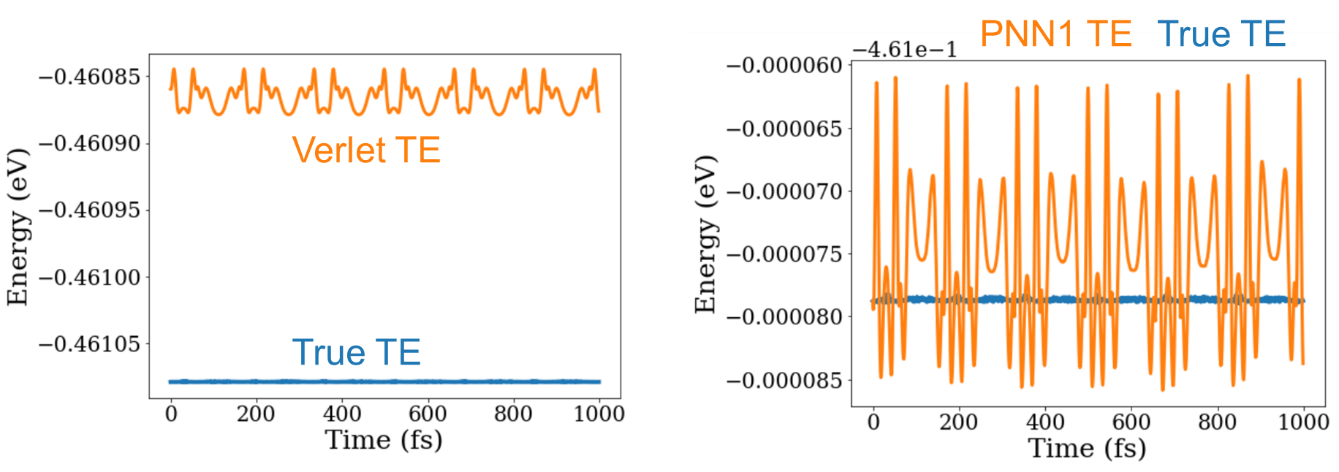
*

*Figure S5: Energy conservation for the Verlet model compared to PNN 1. While both networks conserve energy, PNN 1 attempts to correct for the discrepancy in the force sub-net, predicting the absolute value of the total energy more accurately.*

The second-best network discovered (PNN Model 2) can be shown as follows:


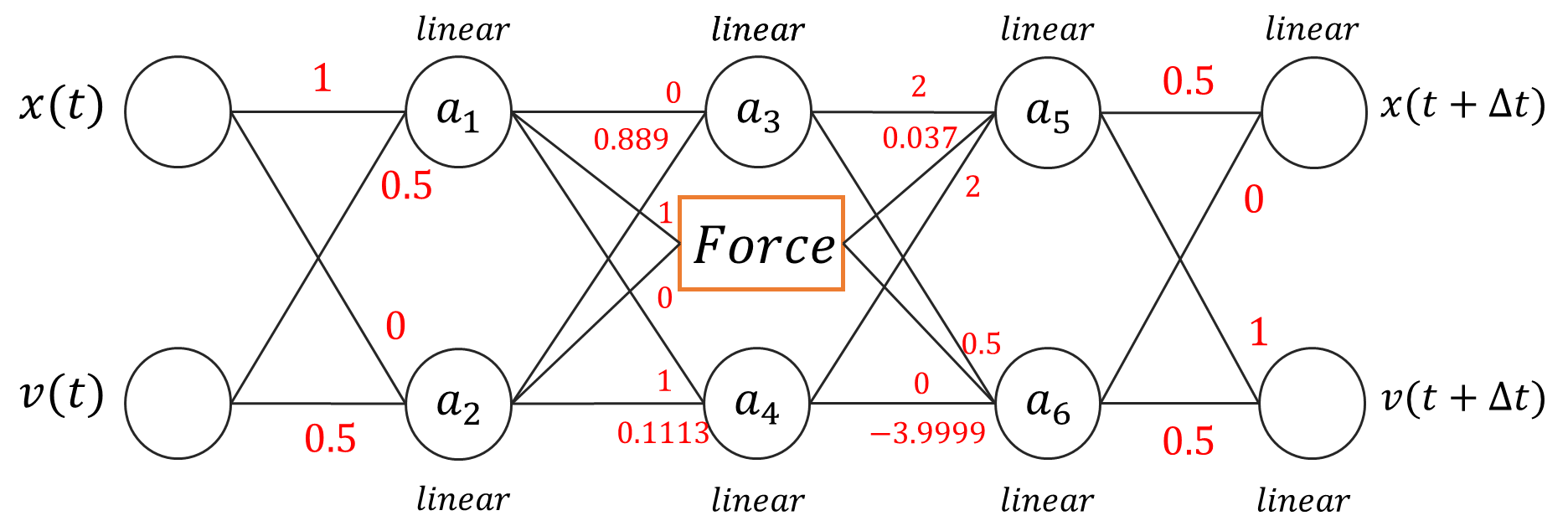


*Figure S6: Activation functions and weights for PNN Model 2*

The RMSE values in this case are (1.87x10^-7^, 6.92x10^-7^), (1.88x10^-7^, 7.07x10^-7^), and (1.51x10^-7^, 5.44x10^-7^) respectively for the training, validation and testing set.

This network can be interpreted as:

| $a_{1}=x(t)+\frac{1}{2}v(t)$ | $a_{2}=\frac{1}{2}v(t)$ |
| --- | --- |
| $a_{3}=0.888a_{2}$ | $a_{4}=a_{1}+0.1113a_{2}$ |
| $a_{5}=2a_{3}+0.037f\left( a_{1} \right)+2a_{4}$ | $a_{6}=\frac{1}{2}a_{3}-3.9999a_{4}$ |
| $x(t+\Delta t)={\frac{1}{2}a}_{5}$ | $v(t+\Delta t)=a_{5}+{\frac{1}{2}a}_{6}$ |

The next best network discovered (PNN Model 3) can be shown as follows:


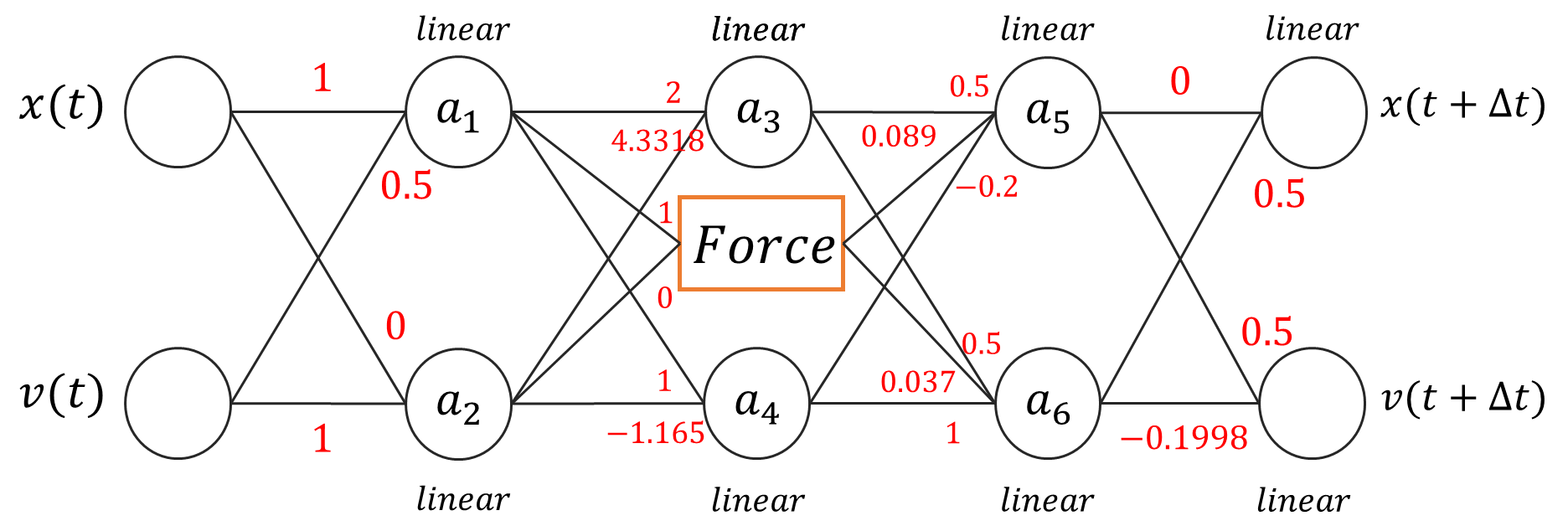


*Figure S7: Activation functions and weights for PNN Model 3*

The RMSE values here are (1.88x10^-7^, 8.25x10^-7^), (1.88x10^-7^, 8.34x10^-7^), and (1.51x10^-7^, 6.81x10^-7^) respectively for the training, validation and testing set.

This network can be interpreted as:

| $a_{1}=x(t)+\frac{1}{2}v(t)$ | $a_{2}=v(t)$ |
| --- | --- |
| $a_{3}=2a_{1}+4.3318 a_{2}$ | $a_{4}=a_{1}-1.165a_{2}$ |
| $a_{5}=\frac{1}{2}a_{3}+0.089f\left( a_{1} \right)-0.2 a_{4}$ | $a_{6}=\frac{1}{2}a_{3}+0.037f\left( a_{1} \right)+a_{4}$ |
| $x\left( t+\Delta t \right)={\frac{1}{2}a}_{6}$ | $v\left( t+\Delta t \right)=a_{5}-{0.1998a}_{6}$ |

From these interpretations, we can construct the outputs $x\left( t+\Delta t \right)$ and $v\left( t+\Delta t \right)$ in terms of the inputs $x\left( t \right)$ and $v\left( t \right)$ and write down the equations below for PNN 2 and PNN 3 (PNN 1 is Eqn 2 and 3 in main manuscript):

$x\left( t+\Delta t \right)=x\left( t \right)+1.0001 v\left( t \right)\Delta t+0.99957 \frac{1}{2}F\left( x\left( t \right)+v\left( t \right)\frac{\Delta t}{2} \right)\frac{\Delta t^{2}}{m}$

$$\begin{aligned} v\left( t+\Delta t \right)=v\left( t \right)+0.99957F\left( x\left( t \right)+v\left( t \right)\frac{\Delta t}{2} \right)\frac{\Delta t}{m} \#\# \end{aligned}$$

$x\left( t+\Delta t \right)=x\left( t \right)+1.000134 v\left( t \right)\Delta t+1.000135 \frac{1}{2}F\left( x\left( t \right)+v\left( t \right)\frac{\Delta t}{2} \right)\frac{\Delta t^{2}}{m}$

$$\begin{aligned} v\left( t+\Delta t \right)=v\left( t \right)+0.99975 F\left( x\left( t \right)+v\left( t \right)\frac{\Delta t}{2} \right)\frac{\Delta t}{m} \# \end{aligned}$$

## S7. Importance of parsimony in discovering equations from data

Section S3 demonstrated that adding a force model to a standard feed forward network is insufficient to discover an accurate integration scheme. In this section, we will demonstrate the importance of parsimony to discover an accurate scheme. We use the generic neural network from Figure 1 in the manuscript and attempt to learn the force sub-net, keeping some weights fixed and some trainable. Specifically, knowing the weights corresponding to a Verlet integrator, any weight that would be designated as “fixed” by the genetic algorithm for this network (weights that have values 0, ½, 1, 2, $\frac{\Delta t}{2}$, $\Delta t$, and 2$\Delta t$) are kept fixed at the values corresponding to the Verlet network, while any weight that is deemed trainable is trained. Our attempt here is to simulate a situation where the genetic algorithm has determined some weights to be fixed, and we wish to understand if the network can learn the force sub-net, and the remaining integrator weights directly from the data. We find that the discovered PNN learns a force sub-net that is a scaling factor times the true force, compensating for this scaling factor by learning the appropriate trainable weight in the integration. This is expected since a raw particle trajectory only provides information about acceleration (force/mass) and not the force and mass individually. We make an error of ~3% in the acceleration learnt by the network, recovering an accurate integration scheme. This shows that by enforcing parsimony, the network can learn the force sub-net to match the training data.

We can also visualize the importance of parsimony via a pareto front of the discovered integration schemes, see Figure S8. We find that PNNs discover integrators that are simple but less accurate than Verlet, and integrators that are more complex but also more accurate.


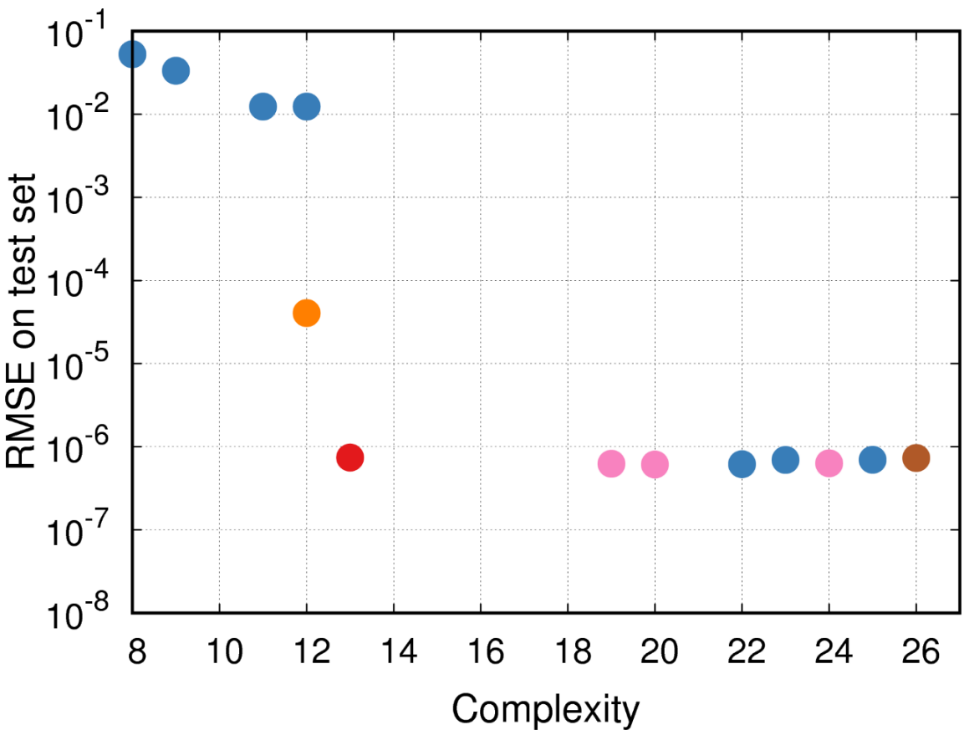


*Figure S8: PNN discovered integration schemes (blue) compared with Verlet integration (red). Also highlighted are PNNs 1 to 3 (pink), PNN 4 (brown), and the Euler integration scheme (orange).*

We highlight one of the integration schemes discovered here, titled PNN 4:

$$x\left( t+\Delta t \right)=x\left( t \right)+1.0001 v\left( t \right)\Delta t+0.9997 \frac{1}{2}f\left( x\left( t \right)+1.03737 v\left( t \right)\frac{\Delta t}{2} \right)\frac{\Delta t^{2}}{m}$$

$$v\left( t+\Delta t \right)=0.99991 v\left( t \right)+0.9991f\left( x\left( t \right)+1.03737 v\left( t \right)\frac{\Delta t}{2} \right)\frac{\Delta t}{m}$$

This integration scheme, while more accurate on one timestep, does not conserve energy over a long time:


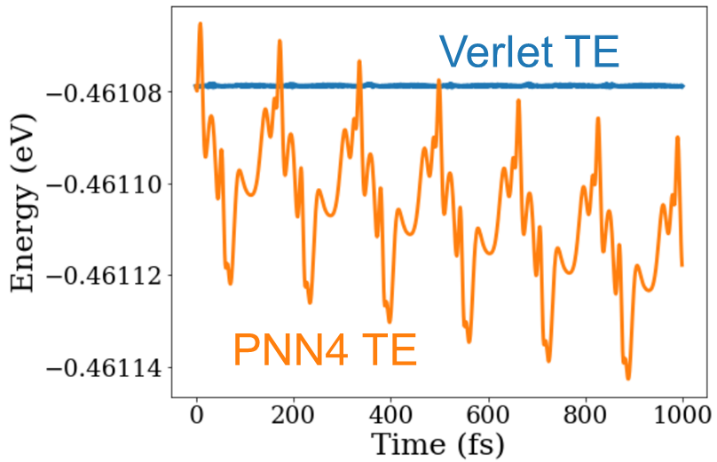


*Figure S9: Energy conservation for PNN 4 compared to the Verlet integration method.*

## S8. Description of training data to discover melting laws

To train our parsimonious neural networks (PNNs) to discover laws that best describe the melting temperature of a wide range of materials, we collected a dataset of experimental melting temperatures for 218 materials (including single elements and perovskites) from the Pymatgen and the Wolfram Alpha repositories respectively. For each of these materials, we collected fundamental material properties such as the bulk modulus $K$, shear modulus $G$, volume per atom, and density $\rho$ by querying the Materials Project repository. We then compute the average sound speed in the material by averaging the S-wave $v_{s}$ and P-wave $v_{p}$ velocities for the material:

$$v_{s}= \sqrt{\frac{G}{\rho}}$$

$$v_{p}= \sqrt{\frac{K+\frac{4}{3}G}{\rho}}$$

$$v_{m}=\left[ \frac{3}{\left( \frac{1}{v_{p}} \right)^{3}+2\left( \frac{1}{v_{s}} \right)^{3}}\text{ } \right]^{\frac{1}{3}}$$

We also compute $a=V_{per atom}^{\frac{1}{3}}$ as an effective interatomic distance, and $m$ to be the mean mass of an atom in the material. Using these quantities, we define four quantities with dimensions of temperature, as follows:

Using these fundamental inputs, we compute four quantities with the dimensions of temperature as follows:

$$\begin{aligned} \theta_{0}=\frac{\hbar v_{m}}{k_{b}a} \#\left( 7 \right) \end{aligned}$$

$$\begin{aligned} \theta_{1}=\frac{\hbar^{2}}{{ma^{2}k}_{b}} \#\left( 8 \right) \end{aligned}$$

$$\begin{aligned} \theta_{2}=\frac{a^{3}G}{k_{b}} \#\left( 9 \right) \end{aligned}$$

$$\begin{aligned} \theta_{3}=\frac{a^{3}K}{k_{b}} \#\left( 10 \right) \end{aligned}$$

where is $\hbar$ the Planck’s constant $h$ divided by 2$\pi$, $a=V_{at}^{\frac{1}{3}}$ is an effective interatomic distance, $m$ is the mean mass of an atom in the material, and $k_{b}$ is the Boltzmann’s constant. We then normalize all inputs and the output (experimental melting temperature) by $\theta_{0}$.

The three dimensionless inputs are thus:

$$\theta_{1}^{'}=\frac{\hbar}{mav_{m}}$$

$$\theta_{2}^{'}=\frac{a^{4}G}{\hbar v_{m}}$$

$$\theta_{3}^{'}=\frac{a^{4}K}{\hbar v_{m}}$$

where is $\hbar$ the Planck’s constant $h$ divided by 2$\pi$. We also normalize the output by $\frac{\hbar v_{m}}{k_{b}a}$ for consistency.

## S9. Parsimonious neural network architecture to discover melting laws

We now design a parsimonious neural network with three inputs $\theta_{1}^{'}$, $\theta_{2}^{'}$, $\theta_{3}^{'}$, one hidden layer with three neurons, and one output, the melting temperature of the material. Each neuron can now take the linear, squared, multiply, inverse, and hyperbolic tangent (tanh) activations. The multiply and inverse activation functions allow for additional complexity in the discovered laws via product and inverse terms. The weights can again be fixed or trainable, with the fixed category allowing weight values of 0 and 1. Unlike discovering integration schemes, the timestep $\Delta t$ is not a relevant quantity anymore, so we do not consider this in our list of fixed weights. This flexibility highlights the ability of PNNs to favor quantities relevant to the underlying physics in comparison to other fixed quantities, as well as arbitrary weights obtained in standard neural network training. Our network thus has twelve weights (each with three possible settings) and four activation functions to optimize. The objective function is now defined to be:

$$F=f_{1}\left( E_{test} \right)+p\left( \Sigma_{i=1}^{4}w_{i}^{2}+\Sigma_{j=1}^{12} f_{2}\left( w_{j} \right) \right)$$

with

$$f_{1}\left( E_{test} \right)={log}_{10} \left( E_{test} \right)$$

$$f_{2}\left( w_{j} \right)=\left\{ \begin{aligned} 0,w_{j}=0 \\ 1,w_{j}=1 \\ 2, w_{j}=trainable \end{aligned} \right.$$

The scores assigned to the linear, squared, multiply, inverse and tanh activations are 0, 1, 2, 3, and 4 respectively. $p$ is a parsimony coefficient that decides the weight on the parsimony terms of the objective function. Increasing $p$ will favor models that are simple, while decreasing $p$ will favor models that are complex but are more accurate.

## S10. Interpreting PNN Model to discover melting laws

We discuss the interpretation of PNN models and their conversion to equations, using the PNN A, B, and C cases mentioned in the manuscript. This is analogous to section S6 above.

The network for PNN A is:


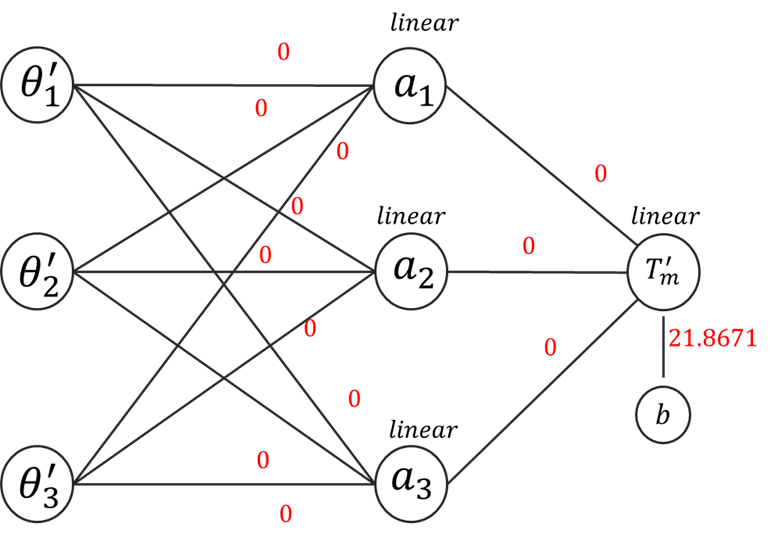


*Figure S10: Activation functions and weights for PNN Model A*

which can be interpreted as:

$a_{1}=0$ $a_{2}=0$ $a_{3}=0$ $T_{m}^{'}=21.8671$

$$T_{m}=T_{m}^{'}\theta_{0}=21.8671 \theta_{0}$$

The network for PNN B is:


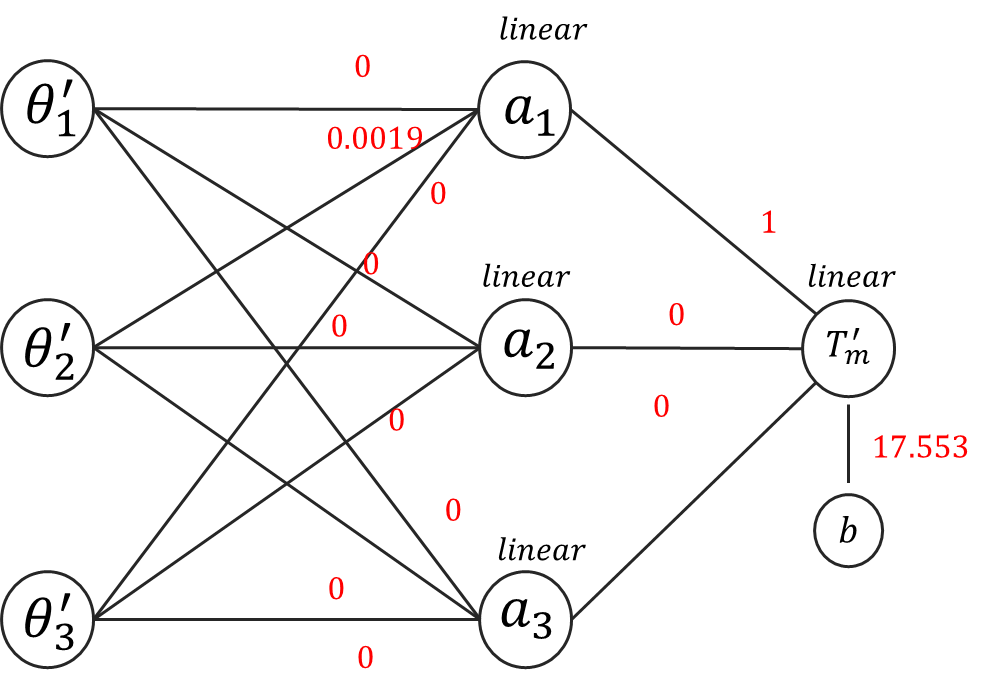


*Figure S11: Activation functions and weights for PNN Model B*

which can be interpreted as:

$a_{1}=0.00198 \theta_{2}^{'}$ $a_{2}=0$ $a_{3}=0$ $T_{m}^{'}=a_{1}+17.553$

$$T_{m}=T_{m}^{'}\theta_{0}=17.553 \theta_{0}+0.00198 \theta_{2}$$

The network for PNN C is:


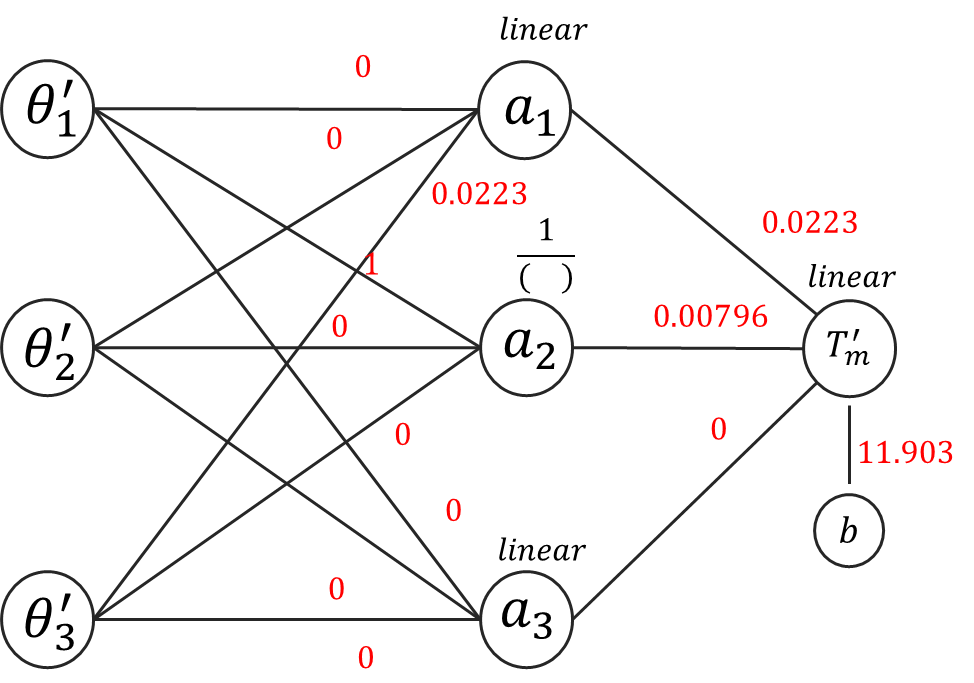


*Figure S12: Activation functions and weights for PNN Model C*

which can be interpreted as:

$a_{1}=0.0223 \theta_{3}^{'}$ $a_{2}=\frac{1}{\theta_{1}^{'}}$ $a_{3}=0$ $T_{m}^{'}=0.0223 a_{1}+0.00796 a_{2}+ 11.903$

$$T_{m}=T_{m}^{'}\theta_{0}=11.9034 \theta_{0}+0.000499 \theta_{3}+0.00796\frac{\theta_{0}^{2}}{\theta_{1}}$$

## References

[1] S. Plimpton, *Fast Parallel Algorithms for Short-Range Molecular Dynamics* (Sandia National Labs., Albuquerque, NM (United States), 1993).

[2] S. Desai and A. Strachan, (2020).

[3] D. P. Kingma and J. Ba, ArXiv Preprint ArXiv:1412.6980 (2014).

[4] F. Chollet, *Keras* (2015).

[5] T. Bäck, D. B. Fogel, and Z. Michalewicz, *Evolutionary Computation 1: Basic Algorithms and Operators* (CRC press, 2018).

[6] F.-A. Fortin, F.-M. De Rainville, M.-A. Gardner, M. Parizeau, and C. Gagné, Journal of Machine Learning Research **13**, 2171 (2012).
